# Deciphering bacterial community changes in zucker diabetic fatty rats based on 16S rRNA gene sequences analysis

**DOI:** 10.18632/oncotarget.10597

**Published:** 2016-07-13

**Authors:** Chunyan Gu, Ye Yang, Hong Xiang, Shu Li, Lina Liang, Hua Sui, Libin Zhan, Xiaoguang Lu

**Affiliations:** ^1^ Basic Medical College, Nanjing University of Chinese Medicine, Nanjing, Jiangsu, China; ^2^ The Second Affiliated Hospital of Dalian Medical University, Dalian, Liaoning, China; ^3^ Department of Emergency Medicine, Zhongshan Hospital, Dalian University, Dalian, Liaoning, China; ^4^ Institute of Integrative Medicine, Dalian Medical University, Dalian, Liaoning, China

**Keywords:** type 2 diabetes, zucker diabetic fatty rat, gastrointestinal microbe, 16S rRNA, microbial communities, Pathology Section

## Abstract

The aim of the present pilot study was deciphering bacterial community changes in Zucker diabetic fatty rats (ZDF rats), a model of type 2 diabetes. Recent studies unmasked that the status of gastrointestinal tract microbiota has a marked impact on nutrition-related syndromes such as obesity and type-2 diabetes (T2D). In this study, samples taken from the gastrointestinal tracts (GI tracts) of ZDF and their lean littermates (ZL rats) were subjected to 16S rRNA gene sequence-based analysis to examine the characteristic bacterial communities, including those located in the stomach, duodenum, jejunum, ileum, cecum and feces. Results revealed that the *Firmicutes/Bacteroidetes* ratio was increased and greater numbers of *Lactobacillus* were detected along GI tracts in ZDF rats compared to ZL rats. In conclusion, this work is the first study to systematically characterize bacterial communities along ZDF rat GI tract and provides substantial evidence supporting a prospective strategy to alter the GI microbial communities improving obesity and T2D.

## INTRODUCTION

The global prevalence of diabetes has risen to be 9% among adults aged over 18 years in 2014 [1]. T2D comprises around 90% of people with diabetes in the world [2]. It is a worldwide concern that more than half a billion people will be affected by this disease by 2030 [3]. An investigation based on sample weighting indicated nearly 113.9 million Chinese adults suffering from diabetes and 493.4 million people with prediabetes [4].

The development of T2D is a complex process implicating synergistic effects of genetic susceptibility and environmental factors [5]. Recent studies have unmasked that the status of gastrointestinal tract microbiota has a marked impact on nutrition-related syndromes such as obesity and T2D [6]. Although genetic factors promote the susceptibility of metabolic disease, the contribution of GI tract microbes as potential partaker in the development of T2D cannot be ignored. Signal molecules produced by microbial metabolism stimulate the pancreatic insulin secretion, improve insulin sensitivity and vary insulin signaling [7], or motivate intestinal gluconeogenesis benefiting energy and glucose homeostasis [8]. A large number of bacterial translocation contribute to insulin resistance by disturbing insulin receptor modification and inhibiting the binding of insulin to its receptor [9]. Recently, the next generation sequencing Illumina Miseq of 16S rRNA gene libraries driven by high-throughput technologies has been used to characterize the diversity of bacterial communities [10]. It is an initially crucial process to assemble short sequences into operational taxonomic units (OTUs) in analyzing metagenomic data [11]. We employed the method above to evaluate whether the properties of different GI sections utilize essential selective pressures on microbiota and play critical roles in the forming of GI microbiota.

Zucker diabetic fatty (ZDF) rats with a missense mutation (fatty, fa) in the leptin receptor gene (LEPR) develop obesity, insulin resistance, and T2D [12-15]. The male ZDF rats present an age-dependent diabetes phenotype, which develop hyperglycemia by 8 weeks of age with serum glucose levels remaining high throughout its life-span [16]. By virtue of these features, the ZDF rat is an attractive experimental model for this study. In this study, we characterized the GI tract microbes of specific pathogen free (SPF) ZDF rats using a high-throughput 16S rRNA sequencing technology.

## RESULTS

### Body weight and blood glucose levels

**Figure 1:**
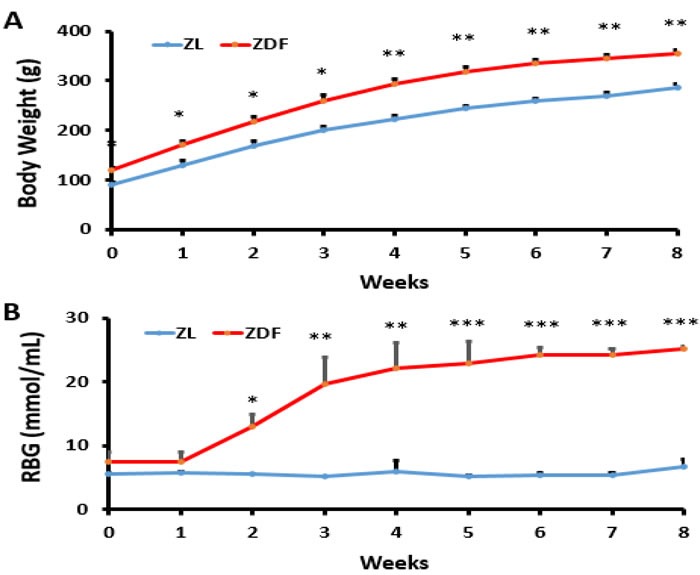
Body weight and blood glucose levels **A**. Body weight of ZDF rats (Zucker diabetic fatty rats) was significantly higher than ZL rats (ZDF-lean rats). *p < 0.05, **p < 0.01, ZDF *vs*. ZL **B.** Random blood glucose of ZDF rats was significantly higher than ZL rats. *p < 0.05, **p < 0.01, ***p < 0.001, ZDF *vs*. ZL**.**

All rats gained weight during the experiment. Body weight of ZL rats was significantly lower at baseline and in the end of this study compared to ZDF rats, and less body weight was gained in the ZL group compared to the ZDF group (Figure 1A). The blood glucose levels were no significant difference between ZL group and ZDF group at first. From week 2, the levels of blood glucose were continuously elevated in ZDF rats (Figure 1B).

### High-throughput 16S rRNA sequencing along the rat GI tract

**Figure 2:**
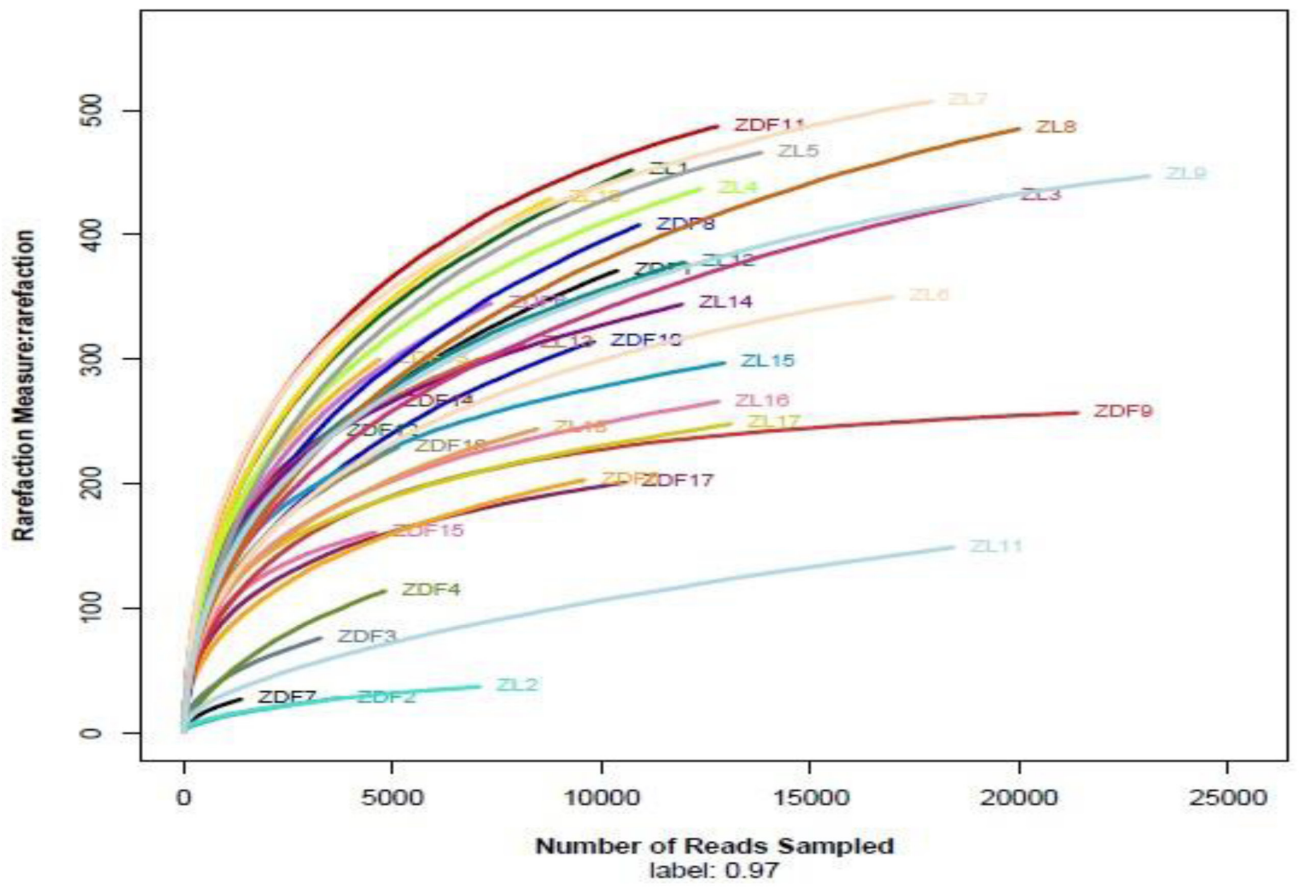
Individual rarefaction curves ZL rats and ZDF rats, *n* = 3, respectively. ZL 1-3, stomach; ZL 4-6, duodenum; ZL 7-9, jejunum; ZL 10-12, ileum; ZL 13-15, cecum; ZL 16-18, feces. ZDF 1-3, stomach; ZDF 4-5, duodenum; ZL 6-8, jejunum; ZL 9-11, ileum; ZL 12-14, cecum; ZL 15-17, feces.

A total of 840,576 sequence reads with a mean length of 344.2 ± 5.49 bp (mean ± SD) were obtained from all samples (stomach, duodenum, jejunum, ileum, cecum and feces) in ZDF rats and the lean littermates (ZL rats) by Miseq Sequencing analysis. Each sample was covered by an average of 24,016 reads. Except one duodenum sample from a ZDF rat containing a very low number of sequences, which was removed from the diversity analysis, all rests met the requirements for library establishment (Table S1 in File S1). The individual rarefaction curves tended to approach the saturation plateau (Figure 2), suggesting high sampling coverage (~99%) was achieved in all samples.

### OTU network analyses of bacterial communities

**Figure 3:**
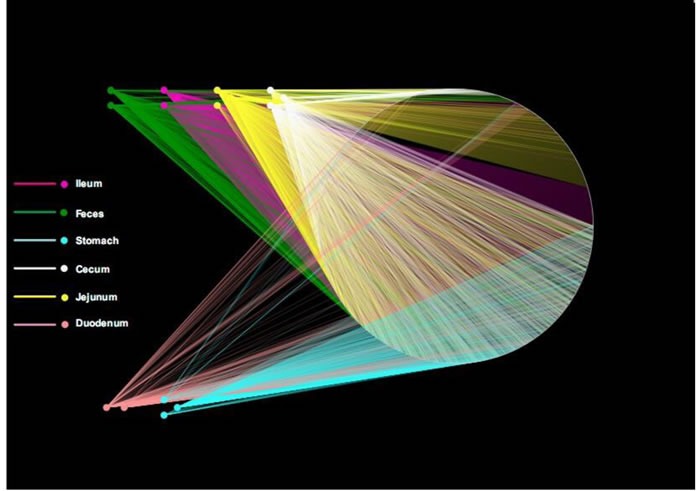
Operational taxonomic unit (OTU) network OTU network analysis of bacterial communities from ZDF rat GI tract samples for the V3 16S rRNA region.

OTUs and different GI tract sites of ZDF rats were labeled as nodes in bipartite network. OTUs were linked with the samples, and their sequences would be found in OTU-nodes [17]. As shown in Figure 3, the OTUs network-based analyses displayed that samples from stomach were more closely related to one another from the adjacent part (duodenum) than that from other anatomic sites, which presented that higher similarity of samples between lower digestive tract and feces than stomach and duodenum. Furthermore, ‘‘shared’’ OTUs were found in the same GI tract site collected from different individuals. Different sites shared different common ‘‘core of flora” both in amount and compositions, which might manipulate unique functions from one GI site to other sites. The stomach and jejunum of the three individuals had a small ‘‘core’’ microbiota (16 and 18 OTUs) composed of bacteria belonging to *Lactobacillus* (Table S2 in File S1), whereas the ileum, cecum and feces of them had a relatively bigger ‘‘core’’ microbiota (81, 79 and 64 OTUs) comprised of bacteria belonging to *Clostridia*, *Bacteroidia*, *Erysipelotrichia* and *Bacilli* (Table S2 in File S1). Different anatomical sites retain their own unique physicochemical environments, including nutrient supplies, pH, redox potential, intestinal motility, and host secretions [18, 19]. OTU network analyses would provide supports for the hypothesis that the properties of different GI sections utilize essential selective pressures on microbiota and play critical roles in the forming of GI microbiota.

### Diversity of the bacterial community along the ZDF rat GI tract

**Figure 4:**
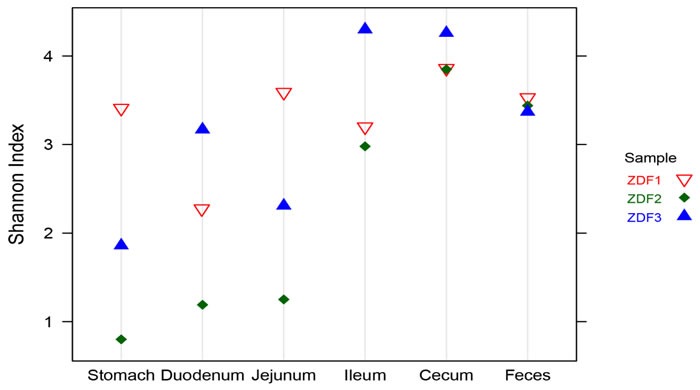
Alpha diversity Shannon diversity of each GI site from three ZDF rats.

To characterize gastrointestinal microbes in ZDF rats, bacterial diversity analysis was performed. For alpha diversity analysis, Shannon Index (SI) was estimated to evaluate the diversity of microbes from each sample (Figure 4). In general, stomach samples had the lowest diversity, while samples from cecum and feces had the highest SI values. In addition, the SI values of stomach and small intestine samples showed much higher inter individual variation than those from cecum and fecal samples.

**Figure 5:**
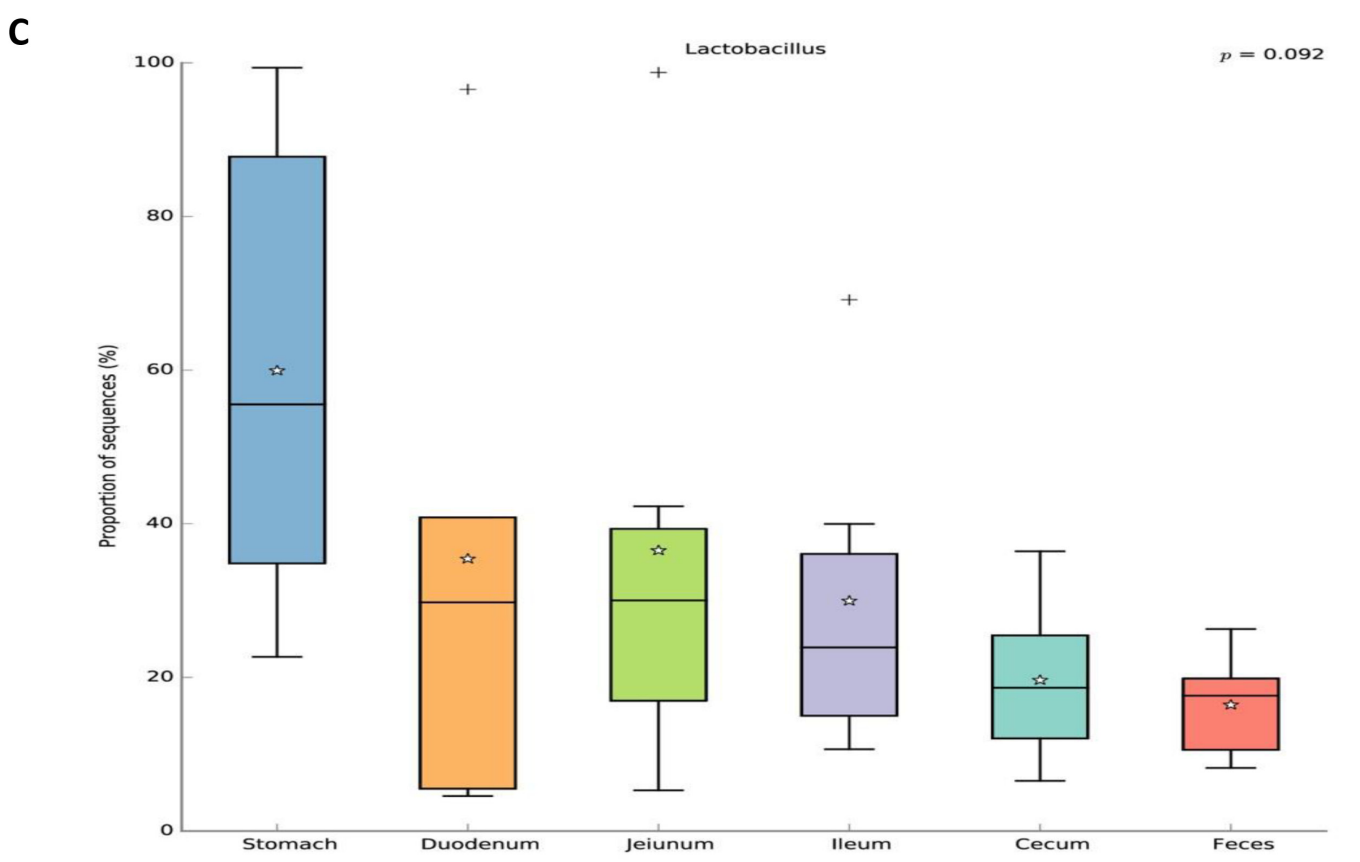
Analysis of Bacterial composition in ZDF rats of each GI site **A**. Beta diversity analysis of the ZDF rat GI tract at phyla level. **B**. Beta diversity analysis of the ZDF rat GI tract at genus level. **C.** Distribution of *Lactobacillus* along the ZDF rat GI tract**.**

For beta diversity analysis of the ZDF rat GI tract, 20 different bacterial phyla were identified. The communities within the distinct sections of the GI tracts differed largely in their compositions and proportions of the major bacteria. The majority of the sequences belonged to *Firmicutes* (68.7%) and *Bacteroidetes* (17.3%), while the rest were *Proteobacteria* (5.9%), *Actinobacteria* (4.0%), *Tenericutes* (3.2%), *Verrucomicrobia* (0.29%) and *unclassified bacteria* (0.63%) (Figure 5). *Firmicutes* was the most abundant phyla in all samples, however, *Bacteroidetes* was the main bacterial phyla of fecal samples (55.4% ± 0.06). In the 6 GI sites, bacterial structure of cecum and fecal samples were similar, but quite different from other anatomic sites. As shown in Figure 5A, the mucosa of the jejunum contained a comparatively higher proportion of *Tenericutes* suggesting that *Tenericutes* may be the new marker for microorganism research in the jejunum of ZDF rat with T2D. At genus level, the facultative bacteria *Lactobacillus*, belonging to *Bacilli* (class), *Lactobacillaceae* (family) was enriched in stomach and small intestine, and decreased from stomach to feces (Figure 5B and 5C). Moreover, genus *Lactobacillus* representing a heterogeneous group has been well documented of immune modulating properties [20] and might potentially contribute to chronic inflammation in diabetic subjects.

### Bacterial taxonomic compositions in ZDF rats compared to ZL rats

**Figure 6:**
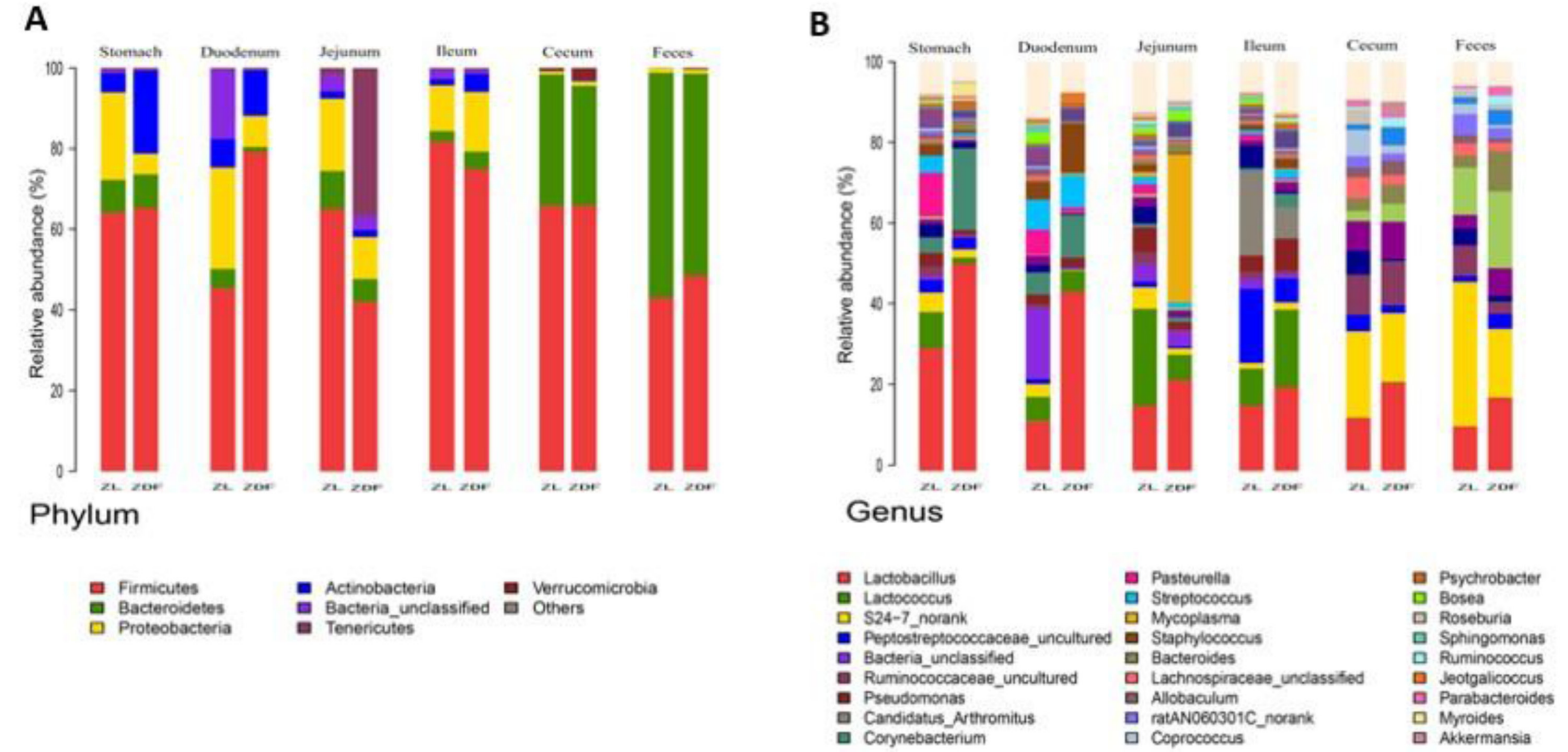
Bacterial taxonomic composition in ZDF rats compared to ZL rats **A**. The difference of Bacterial compositions between ZDF and ZL rats at phyla level. **B**. The difference of Bacterial compositions between ZDF and ZL rats at class level. **C.** Distribution of *Lactobacillus* along the ZDF and ZL rat GI tracts**.**

To explore the effects of missense mutation on the GI tract micro ecology, we compared bacterial communities in different anatomic sites of ZDF rat with its control. At phylum level, in both groups, *Firmicutes* was dominant in stomach and small intestine, and the dominant bacteria of caecum and feces were *Firmicutes* and *Bacteroidetes*. The relative abundance of *Firmicutes* in 5 major functional compartments of the ZDF rat GI tracts and feces were increased (Figure 6A). Although no statistically significant differences were found in the analyzed abundance of the bacterial groups, *Firmicutes* were increased in ZDF group compared to ZL group, while *Bacteroidetes* were decreased in ZDF group compared to ZL group. And the *Firmicutes/Bacteroidetes* ratio increased in ZDF rats compared to ZL rats, especially in Stomach samples (Table 1). At genus level, *Lactobacillus* was increased in ZDF group compared to ZL group and decreased from stomach to feces (Figure 6C).

**Table 1:** Proportion of sequences of *Firmicutes* and *Bacteroidetes* along the gastrointestinal tracts in ZDF and ZL rats

GI tract		Phylum Firmicutes		Phylum Bacteroidetes		Firmicutes/Bacteroidetes ratio	
		Composition	P value	Composition	P value		P value
stomach	ZL	0.69±.099	0.18	0.07±0.03	0.03	11.71±5.61	0.06
	ZDF	0.84±0.25		0.002±0.0008		460.30±299.52	
Duodenum	ZL	0.51±0.23	0.08	0.034±0.033	0.02	25.13±22.51	0.05
	ZDF	0.88±.0.10		0.009±0.001		102.62±22.40	
Jeiunum	ZL	0.67±0.015	0.32	0.21±0.07	0.06	3.41±1.05	0.09
	ZDF	0.74±0.24		0.06±0.03		15.85±9.76	
Ileum	ZL	0.80±.0.17	0.36	0.06±0.02	0.33	13.23±2.80	0.39
	ZDF	0.78±0.10		0.08±0.04		12.06±6.73	
Cecum	ZL	0.65±0.02	0.02	0.43±0.17	0.36	1.65±0.57	0.42
	ZDF	0.60±0.11		0.36±0.11		1.86±1.03	
Feces	ZL	0.34±0.06	0.02	0.65±0.064	0.02	0.53±0.13	0.03
	ZDF	0.43±0.05		0.55±0.06		0.79±0.19	

## DISCUSSION

Microbial community in mammalian gastrointestinal (GI) tract plays an important role in overall health and function. Altering human gut microbiota can influence human health, for instance, dietary changes and antibiotic usage may reduce microbiota complexity [21, 22]. This has led to concern that disruption of gut microbiota could improve conditions such as inflammatory bowel disease, obesity, diabetes, metabolic syndrome, cancer, autoimmune disorders [23]. The human gut microbiome maintains a high intra- and inter-subject variability with four dominant phyla of *Firmicutes, Bacteroidetes, Actinobacteria, and Proteobacteria* [24]. Up to 90% of the gut microbiota belongs to *Firmicutes,* *Bacteroidetes, and Actinobacteria phyla* [25, 26]. Recently, it has been established that the human GM plays crucial roles in type 2 diabetes [27], which demonstrated that a moderate dysbiosis in patients with type 2 diabetes leads to increased risk of a “functional dysbiosis”, rather than a specific microbial species associated directly with T2D pathophysiology.

Numerous metagenomics studies also investigated the gut microbial changes in obesity-the main precursor in the development of T2D in various animal and human studies [28, 29]. Obesity is a major non-communicable global health problem of current era that confers consequential excess risk for T2D [29-31]. Insulin resistance is generally thought to be associated with obesity [32-34]. Typically, T2D is considered to be related to the combination of two metabolic defects, failure of pancreatic beta cells secreting sufficient insulin to compensate for the rising demand and insulin resistance [35, 36]. The latest study reported that GM is a promising modulator of insulin resistance in TLR 2 knockout mice [37]. It is well known that the majority reside microbes in the GI tract modulate host physiology and nutrient intake, and numerous studies focused on the bacterial communities in large intestine and/or feces [38-41].

In order to establish possible associations between the GM changes and T2D, we had conducted the obese diabetic Zucker rat in this investigation, which exhibits hyperglycemia and hyperlipidemia with early onset of insulin resistance. Based on our results, distinctive differences in gut microbiota richness and diversity were observed. In general, stomach samples have the lowest diversity, while samples from cecum and feces have the highest SI values. In addition, the SI values of stomach and small intestine samples showed much higher inter individual variation than those from cecum and fecal samples. Bacterial diversity along the ZDF GI tract was thought to increase from stomach to feces due to stomach and upper small intestine being too harsh (low pH) that makes microorganisms hard to grow and to maintain greater diversity. At phylum level, in both ZDF and ZL groups, *Firmicutes* was dominant in stomach and small intestine, and the dominant bacteria of caecum and feces were *Firmicutes and Bacteroidetes*. The relative abundance of *Firmicutes* in 5 major functional compartments of the ZDF rat GI tract and feces were increased (Figure 6A), in fact, which was also detected in human and mice GI tract [42-45]. Due to limited experimental subjects, the differences of some data are not significant. Although no statistically significant differences were found in the analyzed abundance of the bacterial groups, increased *Firmicutes* and decreased *Bacteroidetes* were examined in ZDF group compared to ZL group. And the rising *Firmicutes/Bacteroidetes* ratio was validated in ZDF rats compared to ZL rats, especially in stomach samples (Table 1). At genus level, *Lactobacillus* was increased in ZDF group compared to ZL group and decreased from stomach to feces (Figure 6C). Our results support the notion that the higher *Firmicutes/Bacteroidetes* ratio and the increased levels of *Lactobacillus* are essential to the prevalence of obesity [46-49]. Similar report claimed that the gut microbiota in T2D patients is characterized by greater numbers of *Lactobacillus* and reduced *Bifidobacterium* species [50]. Our work is consistent with above research findings. As numerous reports focus on fecal samples in animal and human studies of T2D, we for the first time systematically characterizes bacterial communities along ZDF rat GI tract to indicate bacterial communities associated to T2D.

In summary, the present study revealed the characterization of gastrointestinal microbes (GM) in ZDF rat GI tract. Although experimental and clinical studies have shown that targeting gut microbiota might be an effective strategy to prevent and manage diabetes [51-53], the concept of using the microbiota as a biomarker of impending or fully manifest T2D within or outside of the GI tract and for monitoring responses to therapeutic interventions needs to be explored.

## MATERIALS AND METHODS

### Animals

All animal work was performed according to the guidelines of the Institutional Animal Care and local veterinary office and ethics committee of Dalian Medical University (Permit Number: SYXK (Liao) 2008-0002). 5-week-old male ZDF (fa/fa) rats (*n* = 3) and their age-matched normal lean littermate controls (*n* = 3) were purchased from Vital River Laboratories (VRL) (Beijing, China) and housed in the specific pathogen-free (SPF) animal experiment center at Dalian Medical University. The rats were fed with high fat food and water (autoclaved before use) ad libitum and housed at 24 °C ± 2 °C with 65% ± 5% humidity on a 12 h light/dark cycle. Adaptive feeding lasted one week.

### Random blood glucose test

Random blood glucose (RBG) was measured weekly to examine the development of diabetes in the ZDF rats. Glucose levels in tail blood samples were determined using a glucometer (Roche, Mannheim, Germany) from week 0 to week 8.

### Sample preparation

Fresh feces were collected in sterile tube and stored at −80°C immediately. The animals were anesthetized with ether and decapitated before the stomach, duodenum, jejunum, ileum, and cecum were sampled and weighed. Then all samples (36 in total) were snap-frozen in liquid nitrogen and stored at −80°C. The mean lengths of small intestine (including duodenum, jejunum and ileum) and cecum were 6 and 8 cm respectively.

### DNA extraction and PCR amplification

Genomic DNA was extracted from stomach, duodenum, jejunum, ileum, cecum and feces samples using the E.Z.N.A.^®^ Stool DNA Kit (Omega Bio-tek, Norcross, GA, U.S.) according to manufacturer's protocols. The V3 regions of the bacteria 16S ribosomal RNA genes were amplified genomic DNA by PCR (95 °C for 2 min, followed by 27 cycles at 95 °C for 30 s, 55°C for 30 s, and 72°C for 45 s and a final extension at 72 °C for 10 min, 10°C until halted by user) using primers 27F 5′-barcode- AGAGTTTGATCCTGGCTCAG-3′ and 533R 5′-AGAGTTTGATCCTGGCTCAG −3′. 8-bp barcode sequence unique to each samples was attached into forward primer for multiplexing. PCR reactions were performed in triplicate 20 μL reaction mixtures containing 4 μL of 5 × FastPfu Buffer, 2 μL of 2.5 mM dNTPs, 0.4 μL of each primer (5 μM), 0.4 μL of FastPfu Polymerase, and 15 ng of template DNA.

### Illumina MiSeq sequencing

The PCR products were separated by 2% agarose gel electrophoresis and bands of the desired size (approximately 250 bp) were purified using the AxyPrep DNA Gel Extraction Kit (Axygen Biosciences, Union City, CA, U.S.) according to the manufacturer's instructions. Prior to sequencing, the DNA concentration of each PCR product was determined using QuantiFluor™ -ST (Promega, U.S.). The equimolar purified products were pooled and paired-end sequenced (2 × 250) on an Illumina MiSeq platform according to the standard protocols at Majorbio Bio-Pharm Technology (Shanghai, China) and Lingen Biotechnology Co., Ltd. (Shanghai, China).

### Process of sequencing data

Three criteria were followed for demultiplexing and quality-filtering the raw fastq files by QIIME (version 1.17) [54, 55]. Operational taxonomic unit (OTU)-based method was performed to analyze where sequences were split into bins on the basis oftaxonomy and clustered to each bin with the cutoff point of 0.05. UPARSE was used to cluster OTUs with 97% similarity cutoff and UCHIME was applied to identify and remove chimeric sequences. The phylogenetic affiliation analysis of each 16S rRNA gene sequence was introduced by RDP Classifier against the silva (SSU115)16S rRNA database with confidence threshold of 70% [56, 57].

### Diversity analysis

In alpha diversity analysis, Chao 1 and ACE [58] were calculated to estimate community richness. The Simpson index and Shannon index-based measurements were used to evaluate community evenness. Estimators of community richness, evenness and diversity were calculated based on OTUs (97% similarity).

### Statistical analysis

Data are expressed as mean ± SD. Statistical analysis of difference among groups was performed by two-trail Student's test using SPSS Statistics 18.0 (IBM, New York, USA). Variables with non-Gaussian distribution were ASIN-square-root-transformed for comparisons. *P* < 0.05 was considered statistically significant.

## SUPPLEMENTARY MATERIALS TABLES




